# Tislelizumab-induced hyperosmolar diabetic ketoacidosis complicated with rhabdomyolysis in hepatocellular carcinoma patients: case report

**DOI:** 10.3389/fonc.2026.1806333

**Published:** 2026-04-10

**Authors:** Longjun Chen, Xiaojuan Zeng, Tianping Li

**Affiliations:** 1 Department of Pharmacy, West China Hospital, West China Xiamen Hospital, Sichuan University, Xiamen, China; 2 Pediatrics, The Second Hospital of Zhangzhou, Zhangzhou, China; 3 Department of Pharmacy, West China Hospital of Sichuan University, Sichuan, Chengdu, China

**Keywords:** hyperosmolar diabetic ketoacidosis (H-DKA), immune checkpoint inhibitors, immune-related adverse event, PD-1 inhibitors, rhabdomyolysis, tislelizumab

## Abstract

Tislelizumab has been approved for the treatment of various solid tumors. While using it for treatment, it is crucial to pay attention to immune-related adverse events (irAEs), as these often have irreversible consequences for patients. We report a case of a 74-year-old male patient with hepatocellular carcinoma who developed ICI-induced diabetes after receiving tislelizumab treatment. The patient presented with altered mental status, and examination revealed hyperglycemia. He had no prior history of diabetes. Based on laboratory results, he was diagnosed with tislelizumab-induced hyperosmolar diabetic ketoacidosis complicated with rhabdomyolysis. Currently, there are few reported cases of hyperosmolar diabetic ketoacidosis(H-DKA) complicated with rhabdomyolysis caused by immune checkpoint inhibitors, this article aims to clarify the differences in clinical presentation among patients with concurrent rhabdomyolysis and to emphasize the importance for clinicians to identify such immune-related adverse events in patients receiving ICI treatment.

## 1 Introduction

Hepatocellular carcinoma (HCC) is the sixth most diagnosed cancer worldwide and is closely related to chronic liver diseases such as viral hepatitis and alcohol-related liver disease (1), Non-alcoholic fatty liver disease (NAFLD) is rapidly becoming the leading cause (2). Treatment strategies for HCC are becoming increasingly diverse and personalized. The introduction of immune checkpoint inhibitors (ICIs) has reshaped the treatment landscape for advanced HCC, improving objective response rates and overall survival (3). For example, atezolizumab combined with bevacizumab is an established first-line regimen (4).

Tislelizumab, an anti–PD-1 antibody developed in China, is approved for multiple solid tumors and has been applied in advanced HCC (5). Despite its remarkable performance in cancer treatment, tislelizumab’s irAEs should not be overlooked, with hyperglycemia being one of its reported manifestations (6). ICI-induced HHS is a highly challenging emergency; although rare, it progresses rapidly, often presenting clinically as severe dehydration and neurological dysfunction (such as altered consciousness) (7).

This article reports a case of a 74-year-old Asian man with hepatocellular carcinoma who developed vomiting, slurred speech, and altered consciousness after receiving 12 cycles of tislelizumab treatment post-surgery. This case underscores the critical importance of routine blood glucose monitoring during ICI treatment, as well as the necessity for prompt assessment and management of newly developed hyperglycemia. Furthermore patients experiencing hyperglycemic diabetic ketoacidosis (H-DKA) should remain vigilant regarding the potential risk of rhabdomyolysis to avert further serious metabolic emergencies.

## 2 Case report

January 2026, an Asian male was admitted to the hospital due to altered mental status, with symptoms including slurred speech, irrelevant answers, spatial disorientation, inability to recognize family members, accompanied by vomiting, dry mouth, and polyuria. The patient had recently undergone immunotherapy for hepatocellular carcinoma and had a history of hepatitis B surface antigen carrier status and right inguinal hernia.

**Figure 1:**
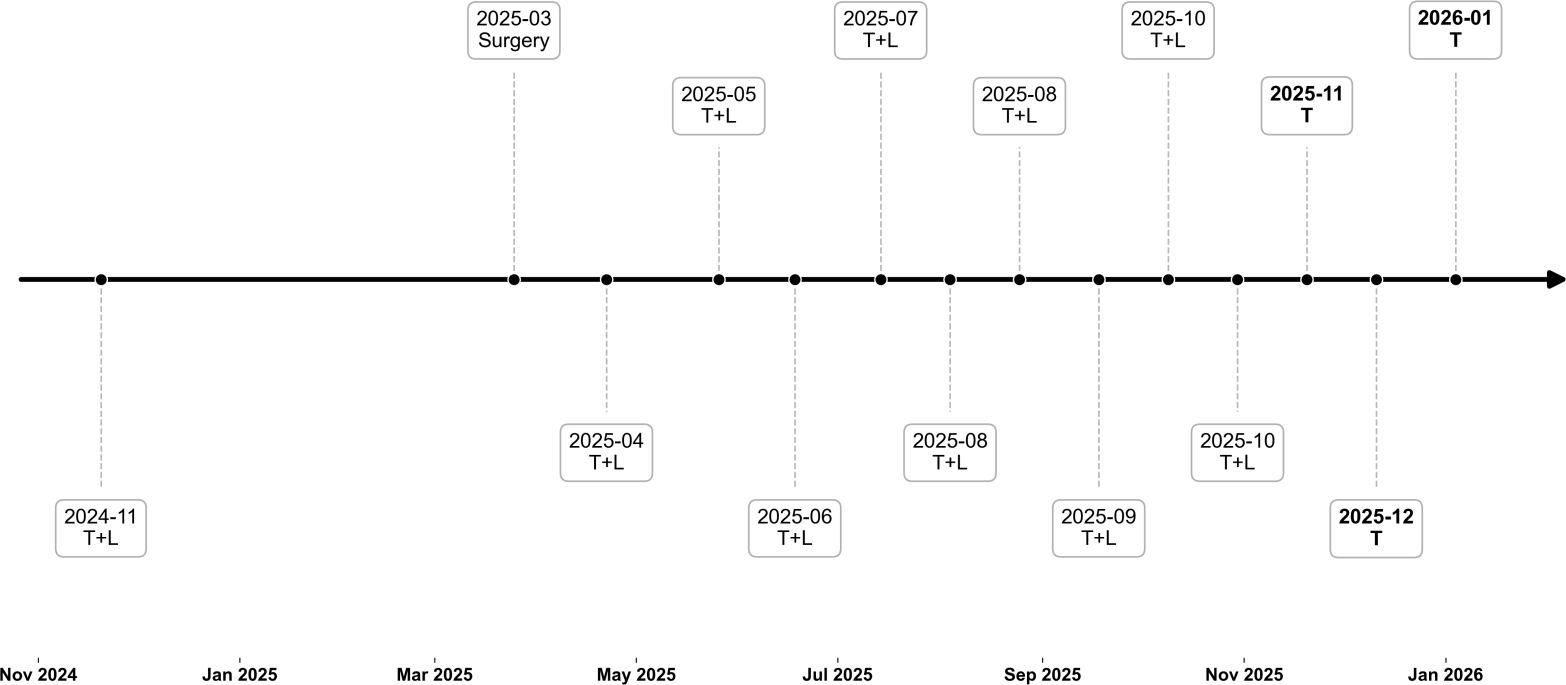
The patient’s anti-tumor treatment process. T, Tislelizumab; L, Lenvatinib. The patient began anti-tumor treatment in late 2024, with an initial regimen of tislelizumab combined with lenvatinib. Subsequent treatment details are unknown until after surgery, when the initial regimen was continued as adjuvant therapy. Later, the patient discontinued lenvatinib due to intolerance-induced diarrhea.

The patient had a history of a bicycle fall 2–3 days prior to this visit. Over a year ago, a blood test revealed the patient was a hepatitis B surface antigen carrier, and entecavir antiviral therapy was initiated. One year ago, the patient was diagnosed with hepatocellular carcinoma and began neoadjuvant therapy with lenvatinib and tislelizumab, during which time interventional liver procedures were also performed. Later, the patient underwent extended right hemihepatectomy and cholecystectomy, with postoperative pathology confirming hepatocellular carcinoma (pT4N0M0 stage IIIB). Postoperatively, the patient continued treatment with lenvatinib and tislelizumab. Due to diarrhea caused by oral lenvatinib, the patient discontinued the drug after the last three cycles and began tislelizumab monotherapy. The patient is allergic to chloramphenicol, manifesting as a rash and itching; has a long history of smoking and alcohol consumption, but no history of diabetes. The patient reported a blood glucose level of 13.4 mmol/L before the last dose of tislelizumab, at which time no appropriate treatment was administered. **Figure 1** illustrates the process of a patient receiving immunotherapy.

The patient was admitted in a delirious state, with blood glucose levels too high to be measured (the glucometer read “HI”). intravenous insulin was initiated to lower blood glucose, and adequate intravenous fluid resuscitation was performed. Laboratory results (**Table 1**) showed a blood glucose level of 65.40 mmol/L, glycated hemoglobin (HbA1c) of 9.0%, and serum β-hydroxybutyrate (β-OHB) was 1.62 mmol/L. Urinalysis showed strongly positive urine glucose and ketones. Blood gas analysis revealed a bicarbonate level of 13.0 mmol/L, anion gap of 28.8 mmol/L, whole blood lactate of 5.96 mmol/L, serum potassium of 5.23 mmol/L, and serum pH of 7.18, supporting a diagnosis of DKA. Simultaneously, the patient’s plasma osmolality rose to 382 mOsm/kg, and bedside blood gas showed a bicarbonate level of 15.50 mmol/L. Based on these clinical data, the patient was suspected of having a HHS. The electrocardiogram revealed a sinus rhythm accompanied by a prolonged QT interval. Cardiac enzyme marker tests indicated elevated levels of creatine kinase (CK) at 16065 IU/L, creatine kinase isoenzyme mass (CK-MB) exceeding 300 ng/ml, myoglobin levels surpassing 3000 ng/ml, and troponin-T measured at 63.8 ng/L. Additionally, the patient exhibited dark brown urine resembling soy sauce, which confirmed the diagnosis of rhabdomyolysis. The kidney function test results showed a creatinine level of 293.14 μmol/L and an estimated glomerular filtration rate(eGFR) of 17 ml/min/m^2^, indicating acute renal failure. Thyroid function profiling indicated a free triiodothyronine (FT3) level of 1.53 pmol/L and a thyroid-stimulating hormone (TSH) level of 0.44 mU/L, a hormonal profile characteristic of central hypothyroidism.

**Table 1:** Laboratory findings.

Lab	Patient	Reference range
Blood glucose (mmol/L)	65.40	4.1-5.6
HbA1c (%)	9.0	4.0-6.0
Serum β-hydroxybutyrate (mmol/L)	1.62	0.02-0.27
Bicarbonate (mmol/L)	13.0	22-27
Anion gap (mmol/L)	28.8	12-20
Whole blood lactate (mmol/L)	5.96	1.0-1.8
potassium (mmol/L)	5.23	3.5-5.2
pH	7.18	7.35-7.45
Plasma osmolality (mOsm/kg)	382	275-295
Crenate kinase (IU/L)	16065	50-310
Creatine kinase isoenzyme mass (ng/ml)	>300	<6.22
Myoglobin (ng/ml)	>3000	<72.0
Troponin-T (ng/L)	63.8	0-14
Creatinine (μmol/L)	293.14	68-108
eGFR (ml/min/m2)	17	56-122
FT3 (pmol/L)	1.53	3.85-6.30
TSH (mU/L)	0.44	0.75-5.60

After initial diagnosis, the patient was transferred to the Intensive Care Unit and continuous insulin infusion and intravenous saline. On the first day of admission, after insulin pump therapy, the blood glucose level dropped to 28.5 mmol/L, and on the second day, the patient’s blood glucose level stabilized at around 10–15 mmol/L. Three days after admission, the patient was conscious, and plasma osmolality and pH had returned to normal. Due to gastrointestinal bleeding, the patient was not allowed to eat, and insulin was continuously infused at 2.5 U/h, with blood glucose and blood gas monitoring, and fluid resuscitation rate controlled. During treatment, insulin autoantibodies were negative, and C-peptide was 0.164 nmol/L. Kidney function did not improve, as reflected by a creatinine level of 270.87 μmol/L and eGFR of 19 mL/min/1.73 m². Six days after admission, the patient’s blood glucose level was relatively stable, remaining between 6-11. Arterial blood gas analysis showed a pH of 7.38, calcium ion 1.09 mmol/L, whole blood sodium 144 mmol/L, whole blood potassium 3.6 mmol/L, blood glucose 12.2 mmol/L, anion gap 17.1 mmol/L, and plasma osmolality 295 mOsm/kg. Renal function testing revealed a creatinine level of 485.24 μmol/L and an eGFR of 7 mL/min/1.73 m². The decline in renal function raised concern for possible concomitant immune-related nephritis. Methylprednisolone therapy was initiated on the same day. Repeated testing of cardiac enzyme markers revealed a decrease in CK-MB to 11 ng/ml and myoglobin to 624 ng/ml. Two days earlier, CK had decreased to 7094 IU/L, while troponin-T was measured at 136 ng/L, indicating significant improvement in rhabdomyolysis. Although the patient’s troponin-T level had increased compared to prior readings, a follow-up echocardiogram demonstrated no significant abnormalities. An electrocardiogram indicated T-wave changes. The patient exhibited no cardiac symptoms, and based on the examination results, myocardial damage was not deemed present. Additionally, further testing for myositis-related antibodies returned negative results.

**Figure 2:**
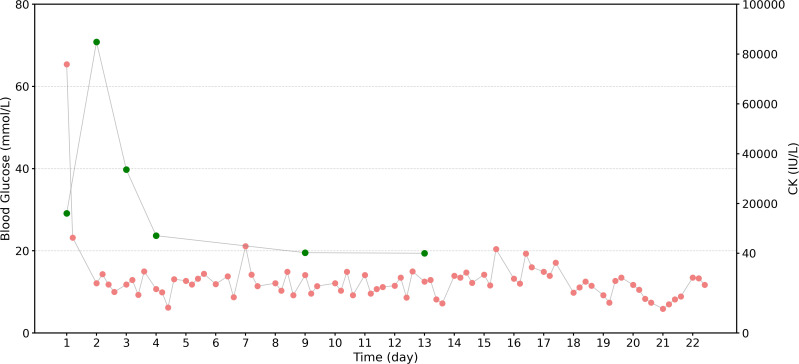
Changes in blood glucose and CK levels during hospitalization. The red scatter points represent the results of blood glucose monitoring four times a day (morning, noon, evening, and bedtime), reflecting blood glucose fluctuations during hospitalization; the green scatter points represent the serum CK level measured simultaneously, reflecting the relief of rhabdomyolysis. It can be seen that CK levels showed a significant decreasing trend with the length of hospitalization, while blood glucose levels generally showed a gradual decrease and tended to stabilize.

On the thirteenth day of hospitalization, the patient discontinued the insulin pump and switched to before meals and at bedtime insulin to control blood sugar (6U aspart insulin injection, ih qn; 10U glargine insulin injection, ih qn). Blood sugar was continuously monitored, and insulin dosage was adjusted accordingly. The patient was subsequently transferred from the intensive care unit to a general ward. During this period, the patient’s blood sugar control was poor, remaining between 13–15 mmol/L. The insulin dosage was adjusted to a maximum of 8U aspart insulin injection, ih qn, and 14U glargine insulin injection, ih qn. After this, the patient’s blood sugar decreased significantly. Subsequently, the insulin dosage was adjusted based on blood glucose levels to stabilize the patient’s blood glucose levels. **Figure 2** illustrates the changes in the patient’s blood glucose level. By hospital day 24, the patient’s renal function had improved significantly. The methylprednisolone dose was being gradually tapered, glycemic control was stabilizing, and discharge planning was underway.

Immune checkpoint inhibitor-induced diabetes has a rapid onset and complex mechanisms. When assessing such adverse reactions, comorbidities, concomitant medications, and disease progression often become confounding factors. The Naranjo’s Causality Assessment Scale (8) provides a structured and standardized assessment framework, quantitatively scoring causality across 10 dimensions. As detailed in **Table 2**, a score of 7 was obtained, suggesting a probable causal relationship.

**Table 2:** Naranjo’s assessment scale of tislelizumab-induced DM.

Scoring items	Scoring criteria (Yes/No/Unknown)	Score of the current case	Scoring evidence
1. Are there previous conclusive reports on this reaction?	1/0/0	1	The product information for tislelizumab clearly states this.
2. Did the adverse event occur after the suspected drug was administered?	2/-1/0	2	The adverse reaction occurred after administration of tislelizumab
3. Was the adverse reaction alleviated when the drug was discontinued or a specific antagonist was administered?	1/0/0	1	After starting insulin therapy, blood sugar levels dropped significantly.
4. Did the adverse reaction reappear when the drug was readministered?	2/-1/0	0	Tislelizumab was not readministered
5. Are there alternative causes (other than the drug) that could have independently caused the reaction?	-1/2/0	2	No other clinical causes of insulin-dependent diabetes mellitus besides drug-related factors have been found.
6. Did the reaction reappear when a placebo was given?	-1/1/0	0	The patient did not receive a placebo
7. Were the drug concentrations detected in the blood (or other fluids) known to be toxic?	1/0/0	0	Undetermined
8. Was the reaction more severe when the dose was increased, or less severe when the dose was decreased?	1/0/0	0	Unadjusted dosage
9. Did the patient have a similar reaction to the same or similar drugs in any previous exposure?	1/0/0	0	The patient denied
10. Was there any objective evidence to confirm the adverse event?	1/0/0	1	The patient had no history of diabetes, but had significantly elevated blood glucose and DKA, with decreased C-peptide levels.
Total score		7	Probable

On assessment of causality, total score ≥9, definitely related; total score 5–8, probably related; total score 1–4, possibly related; total score ≤0, doubtful.

## 3 Discussion

Immune checkpoint inhibitors have become an important treatment strategy in the field of systemic therapy for advanced hepatocellular carcinoma (9). However, not only do immune checkpoint inhibitors exhibit anti-tumor efficacy, but they also cause immune-related adverse effects (10). Endocrine irAEs are a relatively frequent class of toxicities that require lifelong management, and baseline evaluation before treatment and dynamic evaluation during treatment should be emphasized (11). Although the overall incidence of ICI-induced diabetes mellitus(ICI-DM) is not high, once it occurs, it often manifests as absolute or relative insulin deficiency, frequently presenting as diabetic ketoacidosis, and can coexist with HHS, thereby increasing the risk of severe dehydration, altered mental status, and electrolyte disturbances (12, 13).

The onset time of ICI-DM exhibits significant heterogeneity. Most patients develop the disease within weeks to months after ICI treatment, with a median onset time concentrated between 3 and 6 months post-treatment (14). However, some cases show acute onset within one treatment cycle (15), or delayed onset months or even years after discontinuation of treatment (16). In this case, the patient’s onset time exceeded one year after receiving tislelizumab treatment. Mei Dan et al. (17) reported two cases of DKA induced by tislelizumab, which involved two treatment cycles and 6 months of treatment respectively, further demonstrating the difference in onset time. In addition to individual factors, the onset time is also affected by the combination of medications. Studies have shown that the combined use of CTLA-4 therapy may shorten the time to onset of ICI-DM to within three treatment cycles. (15, 18).

This patient was admitted to the hospital due to altered mental status. Based on a comprehensive analysis of the medical history and laboratory test results, the diagnosis was immune checkpoint inhibitor-associated H-DKA. Meanwhile, the patient also had rhabdomyolysis. By questioning the patient and combining the results of laboratory tests to rule out other causes, the rhabdomyolysis was considered to be indirectly caused by adverse drug reactions. Among the adverse reactions associated with tislelizumab, hyperglycemic hyperosmolar state, myositis, and myocarditis can all lead to rhabdomyolysis (19, 20). The patient exhibited no symptoms suggestive of myositis or myocarditis; echocardiography was unremarkable, myositis-specific antibody testing was negative, and although CK was markedly elevated, the CK-MB fraction remained below 5%. Collectively, these findings do not support myositis or myocarditis as the primary etiology. The patient’s thyroid dysfunction was confirmed as central hypothyroidism. Based on the above clinical information, rhabdomyolysis induced by hyperglycemic hyperosmolar state was considered the most likely cause of this episode.

Reports of HHS-inducing rhabdomyolysis are relatively common; however, cases of hyperglycemic crisis complicated by rhabdomyolysis due to immune checkpoint inhibitors are rare. Rhabdomyolysis is a severe complication of HHS that is clinically insidious and often overlooked (21). The combination of H-DKA and rhabdomyolysis not only elevates the risk of acute kidney injury (AKI) but also significantly raises the mortality rate among patients (22, 23). At present, the exact pathogenesis of HHS-induced rhabdomyolysis has not been fully elucidated and is generally considered to be multifactorial. Hyperosmolarity and volume depletion caused by HHS may lead to skeletal muscle ischemia and hypoxia, while hypophosphatemia/hypokalemia and impaired energy metabolism can further aggravate myocellular injury and promote the release of muscle enzymes (24, 25). In the study conducted by Cheng et al. (26), it was demonstrated that patients with HHS-induced rhabdomyolysis presented with older age, as well as higher levels of aspartate aminotransferase (AST), serum creatinine, and blood urea nitrogen, compared with HHS patients without rhabdomyolysis. In the presence of the above risk factors in HHS patients, a high index of suspicion for rhabdomyolysis is warranted, and appropriate relevant diagnostic workup should be promptly performed.

In terms of treatment, after receiving continuous intravenous insulin infusion combined with intravenous fluid resuscitation in the intensive care unit (ICU), the patient’s blood glucose level, osmolality, and acid-base balance gradually returned to normal. This suggests that adhering to standardized hyperglycemic crisis resuscitation procedures remains the core key to successful treatment, specifically including early rapid fluid resuscitation, continuous insulin infusion, close monitoring and correction of electrolyte disturbances (especially potassium regulation), dynamic assessment of osmolality and level of consciousness, and active investigation and treatment of precipitating factors (27, 28). The widely accepted treatment for rhabdomyolysis is aggressive intravenous rehydration (29). In this case, the patient was administered intravenous rehydration using normal saline. Following the treatment, the CK level significantly decreased to the range of 5000–10000 U/L, indicating an improvement in the condition of rhabdomyolysis. During the treatment transition period, the patient still experienced blood glucose fluctuations after switching from a continuous insulin infusion protocol to a basal-meal insulin injection protocol, suggesting that patients with ICI-DM often require more precise insulin titration after the acute phase of disease control, along with enhanced nutritional management and comprehensive management of comorbidities such as renal dysfunction and infection (24, 30). These patients have to rely on insulin for life due to irreversible damage to their pancreatic β cells (31). The long-term blood glucose management and injection burden seriously affect their quality of life.

This patient presented with abnormal blood glucose levels, rhabdomyolysis, and acute renal failure, suggesting a correlation among these three conditions. Both HHS and rhabdomyolysis can cause acute kidney injury. Studies (32) have shown a high prevalence of acute kidney injury (AKI) in HHS patients, and AKI is a significant factor contributing to poor prognosis during hospitalization for HHS patients. A nationwide cohort study in Denmark, analyzing the clinical characteristics of HHS patients, revealed that AKI was a common complication (33). Hyperosmolar state and severe dehydration are the main pathophysiological features of HHS, leading to prerenal acute kidney injury. Furthermore, osmotic diuresis directly caused by hyperglycemia further exacerbates dehydration, creating a vicious cycle (34). Rhabdomyolysis causes acute kidney injury through multiple mechanisms, including myoglobin toxicity, oxidative stress, ferroptosis, inflammatory activation, vasoconstriction, and immune responses, progressing to kidney failure in severe cases (35, 36). The clinical burden of AKI caused by rhabdomyolysis is significant. Large-scale retrospective studies (37) based on the eICU-CRD and MIMIC-IV databases show that the incidence of AKI in patients with rhabdomyolysis is as high as 69.4%, of which 12.1% require renal replacement therapy (i.e., kidney failure). The patient reported in this case also received renal replacement therapy while in the ICU.

The patient’s rhabdomyolysis improved after effective intervention; however, renal function remained impaired, manifested as progressively elevated serum creatinine and persistent proteinuria, raising the possibility of concurrent immune-mediated nephritis. However, the patient also had thrombocytopenia, making renal biopsy a contraindication and preventing definitive diagnosis via renal histopathology. Referring to the CSCO guidelines (38), the patient’s renal injury met the criteria for ICI-related nephrotoxicity grade G3, which recommends permanent discontinuation of ICIs and initiation of prednisone or methylprednisolone glucocorticoid therapy. Based on the highly suspected background of immune-mediated nephritis and in accordance with guideline recommendations, glucocorticoid therapy was initiated for the patient.

For patients receiving tislelizumab therapy, baseline risk stratification and standardized full-course monitoring are critical for reducing the risk of ICI-DM. All patients are required to complete baseline testing of fasting blood glucose, glycated hemoglobin (HbA1c), and islet autoantibodies prior to treatment initiation, to identify high-risk populations including those with pre-existing autoimmune diseases or a family history of diabetes (39). During treatment, fasting blood glucose must be routinely monitored before each cycle of tislelizumab administration, with an intensified monitoring schedule for high-risk individuals. In the case presented here, the patient’s blood glucose level rose to 13.4 mmol/L prior to the final cycle of tislelizumab treatment; however, no targeted intervention was initiated, and immunotherapy was continued, which ultimately led to a cascade of severe adverse events. In addition to routine clinical monitoring, enhanced patient education on symptom recognition is essential (40). If patients develop clinical manifestations including polydipsia, polyuria, nausea, or fatigue (41), urgent testing of blood glucose and blood ketone bodies must be performed promptly to enable early intervention and prevent progression to fatal metabolic crisis.

In this case, we used the Naranjo scale to assess the causal relationship between tislelizumab and new-onset diabetes. The main reasons for selecting this scale are its wide applicability in causal attribution of ADR, ease of use, and ability to improve the reproducibility and comparability of case reports. It should be noted that the Naranjo scale still has distinct limitations when applied to ICI-related adverse reactions. For instance, the long latency period of irAEs, the difficulty in establishing the temporal sequence of some delayed adverse reactions, and the scarcity of clinical data on immune rechallenge, all of which may compromise the accuracy of the scale’s scoring. In the future, building on the core assessment framework of the Naranjo scale and integrating the pathogenesis and clinical characteristics of ICI-related irAEs, we can optimize and develop an ICI-specific ADR causal assessment scale. This will further improve the accuracy of causal determination for immunotherapy-related adverse reactions, and provide more targeted tools for the safety management of ICI treatment.

## 4 Conclusion

Tislelizumab-related H-DKA is a relatively rare yet life-threatening immune-related adverse drug reaction. This type of acute insulin-deficient metabolic crisis can be further complicated by rhabdomyolysis; meanwhile, immune-mediated muscle injury caused by ICI treatment itself can synergistically increase the risk of rhabdomyolysis, and markedly elevate disease severity and the risk of adverse clinical outcomes. Based on previous safety studies and research on tislelizumab-related irAEs, this case report describes a case of tislelizumab-induced H-DKA complicated with rhabdomyolysis. Clinicians should maintain a high degree of vigilance for patients receiving ICI therapy: for those presenting with symptoms including dehydration, vomiting, altered mental status, myalgia, or muscle weakness, blood glucose, blood gas analysis, blood ketones, serum osmolality, CK, and myoglobin should be tested immediately. This facilitates the early identification of metabolic crisis complicated with rhabdomyolysis and improves patients’ clinical outcomes through timely intervention.

## Data Availability

The raw data supporting the conclusions of this article will be made available by the authors, without undue reservation.
